# Novel Multi-Antioxidant Approach for Ischemic Stroke Therapy Targeting the Role of Oxidative Stress

**DOI:** 10.3390/biomedicines12030501

**Published:** 2024-02-23

**Authors:** Camilo Briones-Valdivieso, Felipe Briones, Sofía Orellana-Urzúa, Silvia Chichiarelli, Luciano Saso, Ramón Rodrigo

**Affiliations:** 1 Facultad de Medicina, Universidad Diego Portales, Santiago 8370007, Chile; camilo.briones@mail.udp.cl; 2 Institute for Public Health, Charité-Universitätsmedizin Berlin, Corporate Member of Freie Universität Berlin and Humboldt-Universität zu Berlin, Charitéplatz 1, 10117 Berlin, Germany; fbrionesvaldivieso@gmail.com; 3 Molecular and Clinical Pharmacology Program, Institute of Biomedical Sciences, Faculty of Medicine, University of Chile, Santiago 8380000, Chile; sofiorellana@ug.uchile.cl; 4 Department of Biochemical Sciences “A. Rossi-Fanelli”, Sapienza University of Rome, 00185 Rome, Italy; silvia.chichiarelli@uniroma1.it; 5 Department of Physiology and Pharmacology “Vittorio Erspamer”, Faculty of Pharmacy and Medicine Sapienza University, Piazzale Aldo Moro 5, 00185 Rome, Italy; luciano.saso@uniroma1.it

**Keywords:** stroke, oxidative stress, antioxidants

## Abstract

Stroke is a major contributor to global mortality and disability. While reperfusion is essential for preventing neuronal death in the penumbra, it also triggers cerebral ischemia-reperfusion injury, a paradoxical injury primarily caused by oxidative stress, inflammation, and blood–brain barrier disruption. An oxidative burst inflicts marked cellular damage, ranging from alterations in mitochondrial function to lipid peroxidation and the activation of intricate signalling pathways that can even lead to cell death. Thus, given the pivotal role of oxidative stress in the mechanisms of cerebral ischemia-reperfusion injury, the reinforcement of the antioxidant defence system has been proposed as a protective approach. Although this strategy has proven to be successful in experimental models, its translation into clinical practice has yielded inconsistent results. However, it should be considered that the availability of numerous antioxidant molecules with a wide range of chemical properties can affect the extent of injury; several groups of antioxidant molecules, including polyphenols, carotenoids, and vitamins, among other antioxidant compounds, can mitigate this damage by intervening in multiple signalling pathways at various stages. Multiple clinical trials have previously been conducted to evaluate these properties using melatonin, acetyl-L-carnitine, chrysanthemum extract, edaravone dexborneol, saffron, coenzyme Q10, and oleoylethanolamide, among other treatments. Therefore, multi-antioxidant therapy emerges as a promising novel therapeutic option due to the potential synergistic effect provided by the simultaneous roles of the individual compounds.

## 1. Introduction

Stroke stands as the second-leading contributor to mortality worldwide. With more than 12 million new cases reported in 2019, it represents 11.6% of all deaths. Simultaneously, it is the third-leading cause of combined death and disability, accounting for 5.7% of the total Disability-Adjusted Life Years (DALYs) [1]. The most common type of stroke is ischemic stroke (IS), which is characterised by an occlusion of a vascular structure, leading to acute obstruction of the blood flow to the brain region, for which acute management is based on several therapeutic approaches based on reperfusion, such as thrombolysis or endovascular thrombectomy, with partially improved outcomes [2]. Although this strategy aims to reduce the time of hypoperfusion and, thus, preserve organ function, paradoxically, blood flow restoration leads to relevant additional damage [3]. In the same order of ideas, despite the introduction of successful therapeutic strategies, numerous patients will continue to suffer from physical consequences [4], and, therefore, new approaches to reduce the residual damage are needed. The paradoxical damage generated following the onset of reperfusion is called ischemia-reperfusion injury (IRI), and many potential targets for preventing this injury have been proposed. For instance, reducing the damage induced by oxidative stress (OS) is of major interest [2]. There is a large amount of research outlining the benefits of antioxidant therapies to reduce OS-mediated IRI, translating these models into clinical practice, which has yielded controversial evidence [3]. Within this scenario, it is of paramount relevance to thoroughly comprehend the pathophysiological mechanisms underlying this damage to further understand how new treatment strategies intend to overcome this problem; thus, this review aims to provide an up-to-date summary of the possible contributions of antioxidant compounds for mitigating OS-induced harm in the context of IRI following an IS. In this vein, we explore the hypothesis that using a multi-antioxidant approach for intervening in more than one molecular pathway could improve the benefits of using monotherapies.

## 2. Ischemic Stroke

Following arterial blockade, IS results in a lack of oxygen and glucose deprivation in the brain, affecting both neuronal and glial function, alongside changes in blood vessels and inflammation. Given this oxygen deprivation, neurons suffer from ionic imbalance, altered electron transfer in the mitochondrial oxidative phosphorylation, and membrane depolarisation [5], leading to anoxic depolarisation. Consequently, these events result in the release of neurotransmitters at presynaptic terminals, increasing their concentration and, thus, causing excitotoxicity mainly mediated by glutamate, which cannot be removed due to its energy-dependent removal process [6].

The excitotoxicity is mainly mediated by augmenting the conductance through the N-methyl-D-aspartate receptor (NMDAR). Upon neuronal depolarisation, there is an increase in its conductance, allowing for a large calcium influx into the neurons and triggering an even larger release of calcium from intracellular stores. This increase in the cytosolic calcium concentration is a key step in initiating the intracellular pathways for mitochondrial dysfunction, apoptotic cascade responses, inflammatory responses, and reactive oxygen species (ROS) production, ultimately leading to OS-mediated damage [7].

**Figure 1:**
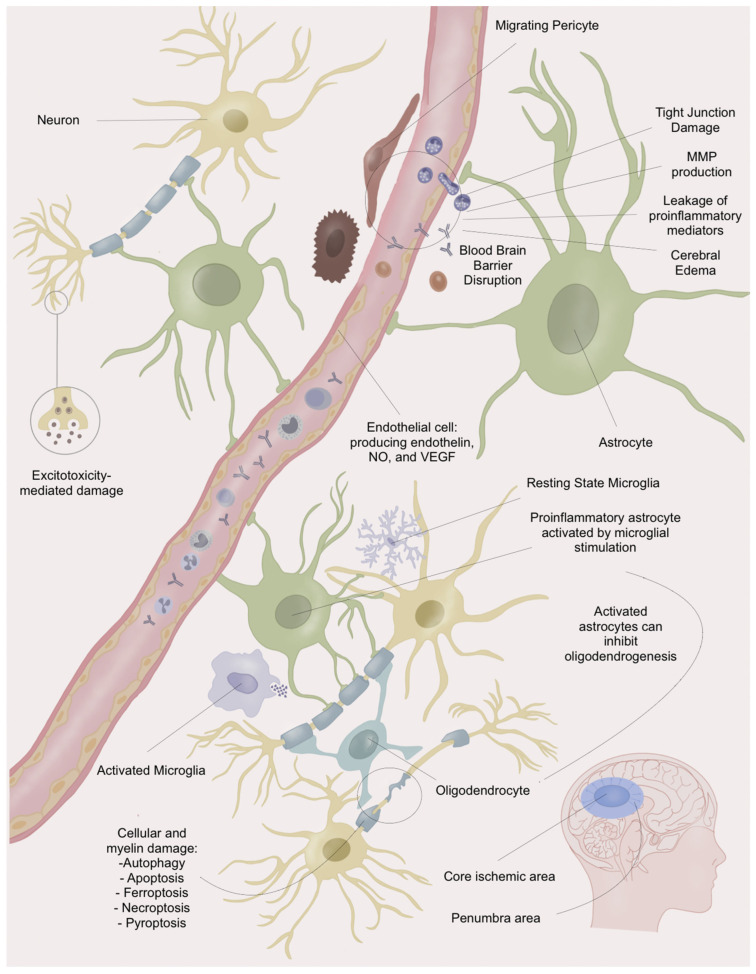
Summary of brain damage mechanisms. MMP: Matrix metalloproteinases. NO: Nitric oxide. VEGF: Vascular endothelial growth factor.

Additionally, after the restoration of blood flow into the brain, the re-entry of oxygenated blood leads to the “oxygen paradox” phenomenon, accompanied by further ROS production, causing greater damage to neurons, astrocytes, oligodendrocytes, and microglia [8]. The entire IRI process, therefore, includes OS-mediated damage by enhancing inflammation and via endothelial dysfunction, leading to the disruption of the blood–brain barrier (BBB); microglial activation; lipid peroxidation; and direct cellular death through ferroptosis, pyroptosis, necroptosis, autophagy, and apoptosis, culminating in potential chronic damage, mainly due to glial scar formation, chronic inflammation, impaired axonal regeneration, impaired remyelination, and impaired neo-angiogenesis [9,10,11,12]. The main mechanisms and molecular pathways involved in brain damage due to IRI following stroke reperfusion are summarised in Figure 1 and Table 1.

## 3. Oxidative Stress

After reperfusion of a blocked cerebral artery, multiple sources contribute to the significant production of ROS, namely as follows:

- Mitochondrial ROS generation;
- Nicotinamide adenine dinucleotide phosphate (NADPH) oxidase enzyme;
- Xanthine oxidase (XO) enzyme.

This leads to hydrogen peroxide production, which can result in the highly harmful hydroxyl anion through the Fenton reaction or the superoxide radical generation. The latter is subsequently dismutated into hydrogen peroxide by various superoxide dismutase (SOD) isoforms. Moreover, several antioxidant enzymes, including catalase, glutathione peroxidase (GPX), and peroxiredoxin (Prx), can effectively degrade hydrogen peroxide [42,43].

### 3.1. Mitochondrial ROS Generation

Mitochondrial ROS generation plays a critical role in IRI. During ischemia, the absence of oxygen leads to mitochondrial dysfunction, resulting in reduced cellular adenosine triphosphate (ATP) production. This dysfunction impairs the activity of the electron transport chain (ETC) within the mitochondria, causing electron leakage and an increase in ROS production. Upon reperfusion, the sudden reintroduction of oxygen to the mitochondria exacerbates ROS generation due to its pre-existing dysfunction. The ETC, responsible for ATP production, becomes a significant source of ROS as electrons leak prematurely and interact with molecular oxygen. This leakage occurs primarily at complexes I and III along the respiratory chain, leading to the overproduction of superoxide anions [19].

### 3.2. NADPH Oxidase

NADPH oxidase (NOX) is a complex oligomeric enzyme that generates ROS by transferring electrons across biological membranes. When activated, NOX undergoes a conformational change that facilitates the assembly of its catalytically active complex. This complex then transfers electrons from cytosolic NADPH to molecular oxygen (O_2_) at the extracellular side of the cell membrane, ultimately leading to superoxide formation [15,16].

### 3.3. Xanthine Oxidase

Xanthine oxidase is an enzyme that plays a role in purine metabolism. It catalyses the conversion of hypoxanthine and xanthine to uric acid, producing ROS as by-products. During ischemia-reperfusion events, XO becomes notably significant in ROS generation due to the increased availability of hypoxanthine and xanthine. Upon restoration of oxygen supply during reperfusion, XO is activated, producing superoxide radicals and hydrogen peroxide [17,18].

### 3.4. Reactive Nitrogen Species

In addition to increased ROS production, there is an augmented generation of reactive nitrogen species (RNS) during IRI. For example, the reaction between the superoxide anion and nitric oxide (NO), locally produced in the brain by type 1 neuronal nitric oxide synthase (nNOS), results in the highly cytotoxic peroxynitrite anion [5]. Two other types of nitric oxide synthase (NOS) contribute to the augmented production of RNS: induced type 2 NOS (iNOS) and type 3 endothelial NOS (eNOS). Activation of eNOS and nNOS are calcium-dependent, whereas activation of iNOS is calcium-independent. Following reperfusion, the nuclear factor kappa B (NF-κB) pathway upregulates iNOS, leading to excessive NO production, which promotes inflammation by increasing the release of proinflammatory factors and altering vascular permeability through the NO/caveolin-1/matrix metalloproteinase (MMP) pathway [12].

Peroxynitrite anion is also responsible for altering protein function through tyrosine nitration. For instance, tyrosine nitration of Kelch-like ECH-associated protein 1 (Keap1) and tumour protein p53 (TP53)-induced glycolysis and apoptosis regulator (TIGAR) has significant implications. Tyrosine nitration of Keap1 promotes an alteration of the cellular antioxidant response by inducing the cytoplasmic nuclear shuttling of the Keap1/nuclear factor erythroid 2-related factor 2 (Nrf2) complex in endothelial cells. This prevents the cells from displaying an adequate Nrf2-mediated antioxidant response [12]. Additionally, tyrosine nitration of TIGAR leads to a jeopardised generation of NADPH, provoking endothelial tight junction damage and, thus, an alteration of the BBB. This alteration is mediated through the downregulation of caveolin-1, further activation of the NOS [13,44], alteration of the E6-associated protein (E6AP)/Prx1 signalling pathways, and subsequent reduction in the antioxidant response, ultimately inducing mitochondrial dysfunction and apoptosis [12].

### 3.5. Excitotoxicity

In an ischemic state, the lack of oxygen provokes neuronal depolarisation and increased glutamate release into the presynaptic space. The excitatory neurotransmitter glutamate, through NMDAR activation, can increase intracellular calcium concentration, which is critical in excitotoxicity, ultimately increasing ROS and RNS production [5,27]. Intracellular calcium overload increases the production of free radicals through several mechanisms: overloading mitochondrial function by disrupting the mitochondrial ETC, activating NADPH oxidase activity, activating nNOS, leading to endoplasmic reticulum stress, and inducing the release of metals, such as iron, from intracellular stores. Metals can participate in the Fenton and Haber–Weiss reaction, promoting the overall production of ROS and RNS [43,45] and directly inducing lipid peroxidation and depletion of antioxidants, leading to membrane damage, loss of membrane fluidity, the formation of reactive by-products—such as malondialdehyde (MDA) or 4-hydroxynonenal—and the activation of inflammatory responses. Ultimately, these processes cause cell damage and death through several mechanisms, such as necroptosis, pyroptosis, apoptosis, ferroptosis, and autophagy [12,23,46].

### 3.6. Lipid Peroxidation

Lipid peroxidation is a process leading to the generation of oxidised lipid products, including lipid peroxides and others, whereby ROS, mainly free radicals, target polyunsaturated lipids, leading to the generation of lipid peroxides and other reactive intermediates. Cell oxygen supply is reduced during ischemia, leading to decreased cellular ATP production and mitochondrial dysfunction. ROS production is abruptly increased in the subsequent reperfusion phase, where oxygen is reintroduced. ROS, especially hydroxyl radicals, can target and attack polyunsaturated fatty acids (PUFAs) in lipid membranes, which are susceptible to oxidation due to their double bonds. The attack on PUFA molecules initiates a chain reaction known as lipid peroxidation, a process that involves the following steps [22,47]:

- Initiation: A free radical, often a hydroxyl radical, subtracts a hydrogen atom from a PUFA, creating a PUFA radical, which can occur through enzymatic and non-enzymatic reactions. In non-enzymatic reactions, Fe is a critical component that triggers the Fenton and Haber–Weiss reactions to generate hydroxyl radicals, anion superoxide, and hydrogen peroxide. In enzymatic reactions, ROS generation is mediated by several enzymes, such as lipoxygenases (LOXs), cyclooxygenases (COXs), and NOX, among others.
- Propagation: PUFA radical reacts with molecular oxygen to form a lipid peroxyl radical. This peroxyl radical can, in turn, react with another PUFA, propagating the chain reaction.
- Termination: The chain reaction is terminated when two radicals react with each other, often forming non-reactive products.

Lipid peroxides are highly reactive and can further contribute to cellular damage. These peroxides can affect the integrity and fluidity of cell membranes in terms of increasing their permeability and disrupting cellular functions, thus leading to the formation of products such as MDA, F2-isoprostanes, 4-hydroxy-2-nonenal (HNE), and oxidised low-density lipoprotein (LDL). The reactive aldehydes can form adducts with cellular proteins and nucleic acids, contributing to cellular dysfunction and amplification of OS by promoting a cycle of free radical formation and further lipid damage [23,48]. Cellular regulatory pathways, such as the GPX4 pathway, typically control lipid peroxidation by converting lipid peroxides into non-toxic forms. However, in ferroptosis, a form of regulated cell death characterised by the iron-dependent accumulation of lipid peroxides, GPX4 inactivation, or inhibition leads to the uncontrolled accumulation of lipid peroxides, contributing to cell death [22].

In this context, Acyl-CoA synthetase long–chain family member 4 (ACSL4) has emerged as a potential target for mitigating brain IRI following stroke, as it has been shown to promote neuronal death by enhancing lipid peroxidation. Additionally, the inhibition of ACSL4 has been demonstrated to reduce proinflammatory cytokine production in microglia, suggesting its role in neuroinflammation regulation for addressing IS-related complications [25]. Furthermore, nuclear receptor coactivator 4 (NCOA4), a cargo receptor for ferritinophagy, serves as a potential target for intervention, as its modulation disrupts the protein–protein interaction between NCOA4 and ferritin heavy chain 1 (FTH1), which leads to a reduction in intracellular ferrous iron availability. This disruption not only blocks ferroptosis, but also prevents the selective autophagic degradation of ferritin, subsequently inhibiting the release of iron and mitigating the promotion of the Fenton reaction, which contributes to lipid peroxidation [26].

### 3.7. Hypoxia-Inducible Factor 1 (HIF-1)

Hypoxia-inducible factor 1 (HIF-1) is pivotal in OS-mediated IRI during stroke. The initial response to reduced oxygen levels activates HIF-1, promoting adaptive cellular mechanisms during ischemia through the transcription of genes in charge of regulating the activities of vascular endothelial growth factor (VEGF), members of the Bcl-2 family and phosphatidylinositol-3-kinase (PI3K)/Akt pathways, which in turn, regulate the activity of NF- κB, ultimately being involved in angiogenesis, glucose metabolism, and cell survival [12,49]. However, HIF-1 stabilisation may yield divergent effects in different cellular compartments of the BBB during stroke, given its regulatory impact on multiple target genes [20]. While HIF-1 activation during ischemia is generally regarded as protective, its role becomes nuanced during reperfusion, as the re-oxygenation triggers an intricate interplay where HIF-1 modulates the cellular response to the ensuing surge in ROS leading to BBB disruption and cellular death. HIF-1’s dual role, serving as a guardian during ischemia yet exhibiting context-dependent effects during reperfusion, underscores the complexity of its impact on cell fate [50], being proposed to specifically target HIF-1 in pericytes to avoid BBB disruption, maintaining its beneficial effects during ischemia [20].

### 3.8. NF-κB

NF-κB is a transcription factor that regulates the expression of genes involved in inflammatory responses, cell survival, and immune reactions. Its activation occurs through two distinct mechanisms [51,52]:

- Canonical pathway (classical): Increased ROS production and pro-inflammatory cytokines stimulate Toll-like receptors (TLRs), tumour necrosis factor (TNF) receptors, and interleukin (IL)-1 receptor, among others. This stimulation triggers the activation of the IκB kinase β (IKKβ) complex, which phosphorylates IκBs, marking them for degradation by proteasomes. Subsequently, NF-κB is released, translocating into the nucleus and initiating gene transcription.
- Non-canonical pathway: This pathway involves a different member of the NF-κB family, p100, which produces the active subunit p52. Specific cytokine family members activate this pathway through the IκB kinase α (IKKα) complex.

Both IKKβ and IKKα interact with other signalling pathways such as the p53, MAP kinase, and IRF pathways, ultimately modulating the gene expression of various factors, including TNFα, IL-1α/β, MHC class I, β2 microglobulin, chemokines such as MCP-1 or MIP-1, adhesion molecules such as ICAM-1, E-selectin, or VCAM-1, pro-apoptotic (Bim, Bax) and anti-apoptotic genes (XIAP, bcl-2), as well as different growth factors [51,53,54]. Excessive or prolonged NF-κB activation can lead to the expression of pro-apoptotic factors, contributing to cell death in the affected brain tissue; thus, its activation is associated with BBB disruption and subsequent inflammatory cell infiltration, exacerbated neuroinflammation, and injury. However, NF-κB activation can also induce the expression of anti-inflammatory factors and antioxidants as part of feedback loops. Its resolution phase involves downregulating NF-κB activity to prevent prolonged inflammation and tissue damage [54]. Given its central role in mediating inflammatory responses, NF-κB has emerged as a potential therapeutic target. Modulating its activity may offer a strategy to attenuate the inflammatory cascade and mitigate the consequences of oxidative stress in ischemia-reperfusion injury [54,55].

### 3.9. Nrf2

Nrf2, a member of a large family of transcription factors, regulates over 200 downstream genes based on the presence of antioxidant response elements (AREs) in the nucleus. Activated by OS, Nrf2 prevents its sequestration from Keap1 and subsequent degradation. Upon activation, the Nrf2-musculoaponeurotic fibrosarcoma (Maf) heterodimer is formed; it binds to the AREs of the DNA [56], upregulating the expression of antioxidant enzymes via the PI3K/Akt, ERK/mitogen-activated protein kinase (MAPK) and NF-κB pathways. This activation prevents apoptosis, helps maintain neuronal metabolic homeostasis in astrocytes, preserves oligodendrocytes and myelin, enhances M2 microglial activation, and inhibits M1 polarisation [21,56,57]. Nrf2 remains activated for days to weeks after the initial ischemic insult, providing lasting effects [56].

Furthermore, Nrf2 promotes the expression of downstream genes and their enzyme production, such as the heme oxygenase-1 (HO-1) enzyme, while also playing a significant role in decreasing the activation of the NLR family pyrin domain containing 3 (NLRP3) inflammasome and the subsequent pyroptosis. Previous studies suggest that activating the Nrf2/HO-1 pathway may effectively prevent the harmful consequences of cerebral ischemia and IRI [40].

### 3.10. Caspases

The regulation of apoptosis involves a delicate balance between pro-apoptotic and anti-apoptotic proteins. Pro-apoptotic proteins, such as caspase-1, caspase-3, and Bax, promote cell death, whereas anti-apoptotic proteins, such as Bcl-2 and Bcl-xL, inhibit apoptosis [58]. During ischemic events, an increased activation of Bax and suppression of Bcl-2 are commonly observed, underscoring the significance of the pro-apoptotic/anti-apoptotic protein ratio in determining cell fate following exposure to related signals. Bax upregulates caspase-9 in brain cells, activating caspase-3, a critical step in the apoptotic cascade [55,59]. On the contrary, Bcl-2 is an anti-apoptotic protein that inhibits the nuclear translocation of pro-apoptotic factors and prevents caspase cleavage [55].

Pro-apoptotic pathways encompass proteins that execute apoptosis (e.g., caspases 3, 6, and 7, Smac/DIABLO), initiate or regulate these pathways (e.g., Bcl-10, MAP kinase p38, and c-Jun N-terminal kinase [JNK]), transcription factors regulating apoptotic protein expression (e.g., E2F1, p53, c-Myc, and GADD153), and other proteins that stimulate apoptosis in specific contexts (e.g., inflammatory caspase 11, glutamate receptor NMDAR2a) [59]. Anti-apoptotic pathways involve proteins such as Bcl-x, oestrogen receptor, calmodulin, p21, Raf1, and MAPK extracellular signal-regulated kinase (MEC) and extracellular signal-regulated kinase (ERK) families, among others [59,60].

## 4. Inflammation

During cerebral IRI, NLRP3 and NLRP1 inflammasomes are central in orchestrating neuroinflammation and pyroptosis through interconnected pathways. The NLRP3 inflammasome, extensively studied in stroke models, responds to danger signals such as ROS generated during ischemia-reperfusion. Activation of NLRP3 leads to increased production of proinflammatory cytokines such as IL-18 or IL-1β [31]. Similarly, NLRP1, another member of the NLR family, contributes to neuroinflammation by forming its inflammasome complex. Upon stimulation, NLRP1 activates caspase-1, which processes IL-1β and IL-18 into their active forms. Furthermore, both NLRP3 and NLRP1 inflammasomes cleave Gasdermin D, a pore-forming protein, ultimately triggering pyroptosis—a highly inflammatory form of cell death characterised by membrane rupture and the release of proinflammatory agents [40,61].

Several investigations propose that various neuroprotective agents mitigate cerebral IRI by suppressing the activation of the NLRP3 inflammasome and subsequent pyroptosis. Targeting these inflammasomes with NLRP1 inhibitors has shown promise, as they hinder inflammasome complex formation and ATP binding. Consequently, NLRP1 inhibitors represent potential novel anti-inflammatory drugs to mitigate neuronal damage and attenuate disease progression in stroke patients. However, further investigation is needed to understand the intricate correlation between NLRP1 and NLRP3 in the central nervous system (CNS) [40,61].

### 4.1. Janus Kinase 2/Signal Transducer and Activator of Transcription 3

The Janus kinase 2/signal transducer and activator of transcription 3 (JAK2/STAT3) pathway in IS pathology exhibit a dichotomous role, necessitating further examination to determine the ideal target molecule for therapeutic intervention.

Phosphorylation levels of both JAK2 and STAT3 increase significantly, predominantly in activated microglia, coinciding with heightened STAT3 expression. This upregulation peaks around 24 h after middle-cerebral-artery occlusion/reperfusion. However, overactivation of STAT3 exacerbates M1 microglial activation and neuroinflammation by promoting the secretion of pro-inflammatory cytokines such as IL-6 and TNF-α [28,62]. Phosphorylated STAT3 plays a pivotal role in upregulating NLRP3 expression, contributing to the formation of the NLRP3 inflammasome complex and triggering pyroptosis. Remarkably, inhibiting the STAT3/mammalian target of the rapamycin (STAT3/mTOR) pathway yields a contrasting effect by promoting M2 microglial activation through the reduction in transmembrane protein plexin A2 expression, highlighting the intricate balance between pro-inflammatory and anti-inflammatory responses governed by the JAK2/STAT3 pathway in IRI [28,63].

In contrast, the JAK2/STAT3 pathway has been reported to exert neuroprotective effects through the activation of the VEGF receptor pathway, promoting cerebrovascular angiogenesis, and through the activation of the PI3K/AKT/mTOR pathway, inhibiting caspase-3-induced neuronal apoptosis in IS [28]. Thus, the dual role of the JAK2/STAT3 pathway in microglial activation and modulation of neuroinflammatory responses underscores its significance in the pathophysiology of IS, warranting further exploration for targeted therapeutic interventions in central nervous system diseases and injuries [62].

### 4.2. Microglia Activation

Microglia, comprising 4–11% of the CNS cell population, serve as the resident macrophages in the CNS [28] and play a significant role in cerebral IRI following a stroke, orchestrating a complex immune response with both beneficial and detrimental consequences. Upon stroke occurrence, microglia are activated in response to damage-associated molecular patterns (DAMPs) recognised by pattern recognition receptors (PRRs) on their cell surface. This activation initiates intracellular signalling pathways, including NF-κB, MAPK, and inflammasome pathways. Consequently, within the first 24 h after stroke onset, microglia undergo a phenotypic shift from a resting state (M0) to a pro-inflammatory M1 phenotype, producing and releasing pro-inflammatory cytokines such as the TNF-α, interferon-gamma (IFN-γ) IL-1β, IL-6, and IL-12 [28,29]. These pro-inflammatory cytokines contribute to neuroinflammation and immune responses in the CNS, potentially exacerbating tissue damage. Additionally, microglia interact with other immune cells, further propagating neuroinflammation and BBB breakdown. The outcome of this initial phase of microglia activation depends on the balance between pro-inflammatory and anti-inflammatory responses [29].

Microglia also exhibit an alternative activation state known as the M2 phenotype, characterised by the expression of anti-inflammatory cytokines such as IL-10, transforming growth factor beta (TGF-β), insulin-like growth factor 1 (IGF-1), and arginase 1 (Arg1) [28], which participate in tissue repair and inflammation resolution. Approximately three days after stroke onset, M2 microglia predominates at the infarct site [54].

The balance between M1 and M2 microglia phenotypes is essential for regulating neuroinflammation and ultimately influencing the extent of tissue damage and repair. This balance can be manipulated via the interferon regulatory factors (IRFs) 5/4 regulatory axis, suggesting that the M1- and M2-subtype balance is regulated in a see–saw-like manner. Moreover, it has been suggested that attenuating M1 activation and enhancing M2 responses of microglia, as well as downregulating the TLR4/NF-κB pathway, represent promising therapeutic targets in IS [54].

## 5. Blood–Brain Barrier Disruption

The BBB is a dynamic barrier composed of brain microvessel endothelial cells (ECs), pericytes, astrocytes, and a basement membrane, which regulates the exchange of molecules between the bloodstream and the brain. Each component plays a crucial role in maintaining the barrier’s integrity and selective permeability [33,34,35,36,37]:

- ECs feature specialised transport systems for selective transcytosis, and tight junctions limit paracellular transport [64].
- Pericytes contribute to BBB maturation and stabilisation, possessing contractile properties that influence blood flow [64].
- Astrocytes, enveloping over 99% of the BBB, provide structural support, regulate blood flow and electrolyte homeostasis, and influence tight junction expression and function. They release factors such as NO and VEGF, impacting vasodilation and oedema. Astrocyte–endothelial cell interactions induce specific phenotypes crucial for maintaining BBB homeostasis, especially during neuroinflammation following IS [37,65].

In addition to the physical barrier formed by tight junctions, the BBB possesses a transport barrier with low rates of endocytosis/transcytosis and a metabolic barrier comprising various enzymes. The endothelial glycocalyx layer and the expression of leukocyte adhesion molecules further modulate the BBB permeability. Equipped with a comprehensive molecular transport system, the BBB maintains brain homeostasis by facilitating essential nutrient transport and preventing toxic compound accumulation [37,65].

The release of ATP from injured neurons attracts immune cells such as neutrophils, monocytes, and T cells to the damage site, while changes in BBB permeability facilitate immune cell infiltration, exacerbating neuroinflammation. This response, coupled with upregulated MMP-9 and p21-activated kinase (PAK) activity, further exacerbates BBB disruption [64,66,67].

In pathological conditions like stroke, BBB integrity is compromised, leading to increased transcytosis and altered cellular interactions. For instance, pericyte loss has been linked to increased endothelial transcytosis without affecting tight junctions. Understanding these mechanisms is essential for developing interventions targeting BBB dysfunction and improving stroke patient outcomes [37].

## 6. Antioxidant Bioactive Molecules against Ischemic Stroke

**Figure 2:**
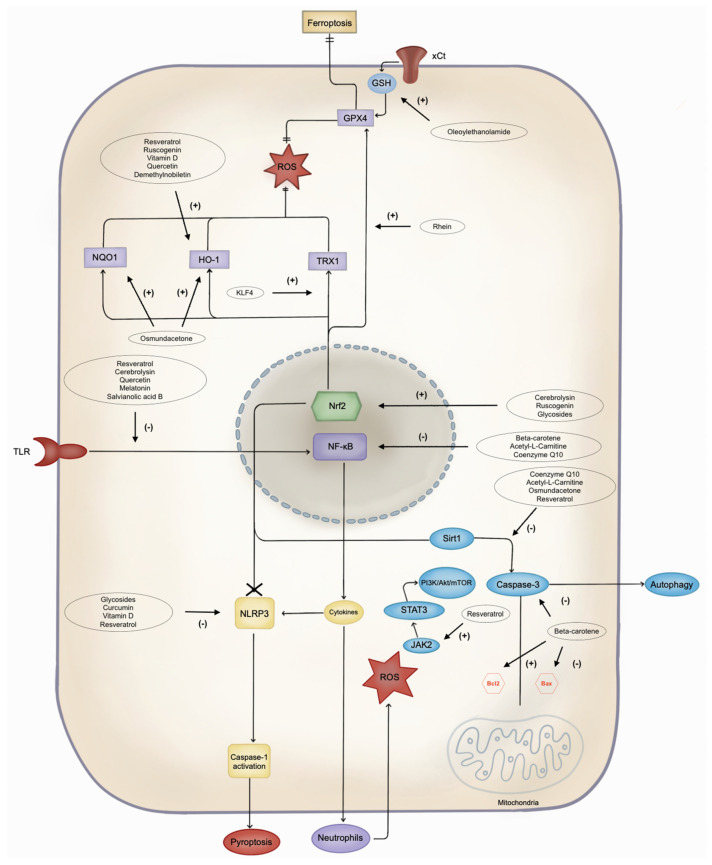
Pathways of cell damage and possible antioxidant target. NLRP3: Leucine-rich repeat protein 3; ROS: reactive oxygen species; JAK2: Janus kinase 2; STAT3: signal transducer and activator of transcription 3; PI3K: phosphoinositide 3-kinase; Akt: protein kinase B; mTOR: mammalian target of rapamycin; Bcl2: B-cell lymphoma 2; Bax: Bcl-2-associated X protein; Sirt1: silent information regulator 1; NF-κB: nuclear factor-kappa B; Nrf2: nuclear factor erythroid 2-related factor 2; TLR: Toll-like receptor; DHBAc: dihydroxybenzoic acid; KLF4: Krüppel-like factor 4; TRX1: thioredoxin 1; HO-1: heme oxygenase-1; NQO1: NAD(P)H quinone oxidoreductase 1; GPX4: glutathione peroxidase 4; GSH: glutathione; xCt: cystine/glutamate antiporter.

Multiple antioxidant molecules have been described to exert beneficial effects after IRI following a stroke. Table 2 and Figure 2 summarise their features and potential therapeutic targets.

### 6.1. Polyphenols

Polyphenols comprise a diverse group of antioxidants, sharing a common feature of containing at least one aromatic ring with multiple hydroxyl groups [54]. Some polyphenols, such as resveratrol [68], curcumin [69], and quercetin [70], have been studied to assess their neuroprotective effects, and several mechanisms have been implicated.

Although until now, resveratrol has been used to treat some forms of cancer, inflammation, diabetes, and myocardial IRI, among other diseases, the neuroprotective effects of resveratrol have been demonstrated through its antioxidant function (upregulate HO-1 and SOD), anti-inflammatory (downregulate TLR4), and antiapoptotic (reduce the activity of caspase-3) effects. Moreover, resveratrol’s neuroprotective potential could also be attributed to regulating the JAK/STAT pathway [68].

Curcumin exerts direct protective effects against cerebral ischemia by multiple mechanisms, such as inhibiting mitochondrial-induced apoptosis and endoplasmic reticulum stress while stimulating neurogenesis. Indirectly, it fosters neuroprotection by shifting microglia polarisation from the proinflammatory M1 state to the anti-inflammatory M2 state [69]. Additionally, curcumin demonstrates anti-inflammatory effects by inhibiting the NLRP3-inflammasome [69].

Similarly, quercetin offers protective effects by inhibiting autophagy and acting as an antioxidant through HO-1 upregulation [70]. Furthermore, it facilitates microglia M2 polarisation by modulating the PI3K/Akt/NF-κB signalling pathway [70,71].

### 6.2. Carotenoids

Carotenoids, including β-carotene (βCAR), are known for their potent antioxidant properties, exerting neuroprotective effects against cerebral IRI. βCAR demonstrates neuroprotection by inhibiting caspase-dependent apoptosis, evidenced by the downregulation of Bax and upregulation of Bcl-2 mRNA expression. Additionally, it exerts anti-inflammatory effects by inhibiting NF-κB expression, resulting in decreased production of pro-inflammatory cytokines such as TNF-α, IL-6, IL-1β, as well as COX-2, and iNOS [55]. Another noteworthy carotenoid, astaxanthin (ATX), structurally similar to βCAR, has consistently shown promising results in reducing cerebral infarction size and caspase-3 activity in rat models of cerebral ischemia [55]. ATX, being lipid-soluble, boasts a broad spectrum of pharmacological effects, including anticoagulant, anti-inflammatory, and antioxidant properties. Interestingly, ATX can penetrate the BBB, exhibits low toxicity, and has higher antioxidant activity than other carotenoids such as α-carotene, βCAR, lycopene, and lutein. Moreover, ATX has been associated with the enhancement of SOD1 and SOD2 expressions, indicating its potential to promote antioxidant defence mechanisms. However, further investigation into its impact on markers of Nrf2 activations is warranted [72].

### 6.3. Vitamins

Vitamins represent another interesting group due to their antioxidant properties and potential neuroprotective effects. For instance, studies have indicated that high-dose supplementation of 1,25-vitamin D3 can mitigate cerebral damage following a stroke [40]. Although the precise regulatory mechanism by which vitamin D confers protection against cerebral IRI remains elusive, it is suggested that vitamin D may activate the Nrf2/HO-1 antioxidant pathway while suppressing the NLRP3-mediated pyroptotic pathway [40].

Moreover, supplementation of folic acid, a type of vitamin B essential for nervous system development and function, holds promise in stroke prevention and as a potential treatment to ameliorate ischemic injury-induced cognitive decline. Folic acid supplementation may achieve this by inhibiting excitotoxicity, as evidenced by the downregulation of NMDAR expression [73].

Additionally, all-trans retinoic acid (ATRA), an active metabolite of vitamin A, has been investigated for its potential to preserve BBB integrity. Promising findings suggest that ATRA administration inhibits JNK and P38 phosphorylation while also reducing MMP-9 content, indicating its potential to safeguard BBB integrity [67].

### 6.4. Hormones

Due to its low toxicity, melatonin, a hormone mainly produced and secreted by the pineal gland, has undergone extensive study for its protective effects against various neurological disorders, including IS. Melatonin administration has demonstrated protective effects against cerebral IRI by mitigating excitotoxicity, OS, endoplasmic reticulum stress, mitochondrial dysfunction, and BBB injury. Additionally, melatonin has been shown to reduce glial activation, inflammasome formation, pyroptosis, and necroptosis by downregulating the high-mobility group box protein 1 (HMGB1)/TLR4/NF-κB signalling pathway [74]. In light of these findings, a recent pilot clinical study evaluated melatonin’s potential efficacy in treating acute IS patients unable to receive reperfusion therapy, yielding promising results for functional improvement and neurological recovery [75].

Studies involving other hormones have demonstrated that administering oestrogen and progesterone after brain ischemia protects against glutamate neurotoxicity, likely through modulation of glutamate transporter expression, thereby enhancing glutamate re-uptake [76]. Additionally, erythropoietin exhibits a neuroprotective role in experimental models of ischemia/reperfusion, hypoxia-ischemia, subarachnoid haemorrhage, and cerebral infarction by activating STAT, which plays a crucial role in neuronal survival and anti-apoptosis [77].

### 6.5. Others

Several other potential neuroprotective antioxidants and their mechanisms are summarised in Figure 2 and Table 2. Notably, acetyl-L-carnitine and coenzyme Q10 (CoQ10) have emerged as promising drugs for mitigating IS damage [78,79].

Acetyl-L-carnitine is an antioxidant derived from carnitine, widely distributed in mammalian tissues, especially in the liver and skeletal muscles. It has demonstrated potential in inhibiting atherosclerosis by regulating blood lipids and suppressing OS and inflammatory gene expression [80]. A recent pilot clinical trial evaluated its efficacy in treating acute IS patients ineligible for reperfusion therapy, revealing promising outcomes. It was suggested that acetyl-L-carnitine’s neuroprotective activity may stem from its regulation of inflammation and OS, as evidenced by significant declines in serum levels of TNF-α, ICAM-1, IL-6, and NSE, along with substantial increases in serum levels of SOD, total antioxidant capacity (TAC), and GPX in treated patients [79].

CoQ10, recognised for its potent antioxidant effects on mitochondrial and lipid membranes, has shown promise as a neuroprotective agent [81], and its role in modulating the expression of genes involved in inflammation and apoptosis pathways is well documented [82]. Past studies have assessed the potential therapeutic role of CoQ10 for preventing dopaminergic neuron degeneration in the context of Parkinson’s disease and thus suppressing the progression of this disease [81]. Furthermore, due to the role of CoQ10 as a free radical scavenger in IS [82], it has been clinically studied for improving outcomes in patients suffering from acute IS, where CoQ10 supplementation significantly improved the National Institutes of Health Stroke Scale (NIHSS) and mini mental state examination (MMSE) scores. However, no significant differences were found in the modified Rankin scale (mRS) score, MDA, SOD, and GFAP compared to placebo [78].

In animal models, several other substances have proven to be effective, for instance, the following:

- Salvianolic acid B (Sal B), a hydrophilic caffeic acid derived from Salvia miltiorrhiza, has been widely studied due to its antioxidative, anti-inflammatory, and neuroprotective properties, probably mediated by blocking the TLR4, p-p38 MAPK, p-JNK, IL-1β, and NF-κB pathways [83].
- Rhein, an anthraquinone, exerts neuroprotective effects by regulating the NRF2/SLC7A11/GPX4 pathway, inhibiting ferroptosis during IRI following a stroke in murine models [84].
- Osmundacetone [85], a natural antioxidant, and Ruscogenin [86], a steroidal sapogenin, have shown effectiveness in reducing the IRI in stroke models in rats by increasing the concentration of Nrf2 mRNA, HO-1, and NQO1. Osmundacetone has also decreased Keap1 and caspase 3 [85].
- Crebabine, an alkaloid with neuroprotective effects, was shown to be effective in a murine model of stroke, reducing cerebral damage by suppressing NADPH and NOX2 activity and through the inhibition of the NF-κB and MAPK pathways [87].
- Glycosides, derived from the Buyang Huanwu Decoction, exert a neuroprotective effect in murine stroke models by reducing pyroptosis by regulating the Nrf2 pathway [88].
- The Krüppel-like factor 4 (KLF4) is a transcription factor related to several cell processes, such as cell proliferation and apoptosis. In murine models, its administration as a recombinant human KLF4 protein has been shown to effectively reduce cerebral IRI’s brain damage by inhibiting cellular oxidative stress through the Nrf2/Trx1 pathway [89].
- Cerebrolysin is a mixture of neuropeptides that, through the inhibition of the TLRs/NF-kB/cytokines pathways and the activation of the Keap1/Nrf2 pathway, has shown to be neuroprotective in murine models of cerebral IRI [90].

**Table 2:** Examples of potential antioxidants for neuroprotection and pathways involved.

Family	Drug	Results	Type of Model	Ref.
Polyphenols	Resveratrol	Nrf2: Upregulate HO-1 and SOD [91] NF-κB: Downregulate TLR4 [92] Sirt1: Downregulate caspase-3 activity [93] Upregulate JAK, ERK, and STAT [94] Upregulate ERK and CREB [95] Upregulate of BDNF/TrkB signalling pathway [96]	Rat models of cerebral ischemia/reperfusion injury summarised through a meta-analysis [68]	[68]
	Curcumin	Downregulate NLRP3 inflammasome	Rat models of cerebral ischemia/reperfusion injury	[69]
	Quercetin	Nrf2: Upregulate HO-1 Downregulate autophagy Upregulate PI3K/AKT/mTOR pathway	Rat models of cerebral ischemia/reperfusion injury	[70]
	Demethylnobiletin (polymethoxy-flavanone)	Nrf2: Upregulate HO-1	Rat models of cerebral ischemia/reperfusion injury	[66]
Carotenes	Beta-carotene	NF-κB: Downregulate caspase-3, and Bax Upregulate Bcl-2 expression	Rat models of cerebral ischemia/reperfusion injury	[55]
	Astaxanthin	Upregulate expression of SOD1 and -2	Gerbil models of cerebral ischemia/reperfusion injury	[72]
Vitamins	Vitamin D	Nrf2: Upregulate HO-1 Downregulate NLRP3-mediated pyroptosis	Rat models of cerebral ischemia/reperfusion injury	[40]
	Folic acid	Downregulate neurotoxicity by downregulation of NMDAR expression	Rat models of cerebral ischemia/reperfusion injury	[73]
	ATRA	Downregulate the JNK/P38 MAPK pathway	Rat models of cerebral ischemia/reperfusion injury	[67]
Hormones	Melatonin	Downregulate the HMGB1: modulates pyroptosis and necrosis Modulation of the TLR4/NF-κB signalling pathway: Upregulate anti-inflammatory mediators MAPK regulation: Downregulate apoptosis	Obese rat models of cerebral ischemia/reperfusion injury	[74]
	Oestrogen Progesterone	Decrease neurotoxicity by modulating glutamate transporter expression and inducing glutamate re-uptake	Rat models of cerebral ischemia/reperfusion injury	[76]
	Erythropoietin	Upregulate STAT	Rat models of cerebral ischemia/reperfusion injury	[77]
Other	Coenzyme Q10	NF-κB: Downregulate p65, TNF-α, and IL-6 Downregulate caspase-3 apoptosis	Rat models of cerebral ischemia/reperfusion injury	[97]
	Acetyl-L-carnitine	Suppress excitotoxicity NF-κB: Downregulate p65, TNF-α, and IL-6 Downregulate caspase-3 apoptosis	Rat models of cerebral ischemia/reperfusion injury	[97]
	Salvianolic acid B (Sal B)	Downregulate TLR4, NF-κB, and IL-1β	Mice models of cerebral ischemia/reperfusion injury	[83]
	Rhein	Nrf2/SLC7A11/GPX4 axis: Inhibit ferroptosis	Rat models of cerebral ischemia/reperfusion injury	[84]
	Osmundacetone	Nrf2: Upregulate HO-1 and NQO1 Downregulate caspase-3 pathway	Rat models of cerebral ischemia/reperfusion injury	[85]
	Ruscogenin	Nrf2 pathway	Mice models of cerebral ischemia/reperfusion injury	[86]
	Crebanine	Downregulate oxidative stress and neuroinflammation mediated by NOX2 in microglia	Rat models of cerebral ischemia/reperfusion injury	[87]
	Glycosides	Nrf2: Downregulate pyroptosis	Rat models of cerebral ischemia/reperfusion injury	[88]
	KLF4	Nrf2: Trx1 pathway	Rat models of cerebral ischemia/reperfusion injury	[89]
	Cerebrolysin	Downregulate TLR/NF-κB/cytokines Upregulate the Keap1/Nrf2/antioxidant signalling pathway	Mice models of cerebral ischemia/reperfusion injury	[90]

Akt: Protein kinase B; BDNF: brain-derived neurotrophic factor; CREB: cAMP response element-binding protein; ERK: extracellular signal-regulated kinase; GPX: glutathione peroxidase; IL: interleukin; JAK: Janus kinase; JNK: c-Jun N-terminal kinase; Keap1: Kelch-like ECH-associated protein 1; HMGB1: high-mobility group box protein 1; HO-1: heme oxygenase-1; MAPK: mitogen-activated protein kinase; mTOR: mammalian target of rapamycin; NLRP3: NLR family pyrin domain containing 3; NtabMDAR: N-methyl-D-aspartate receptor; Nrf2: nuclear factor erythroid 2-related factor 2; NF-κB: nuclear factor kappa-light-chain-enhancer of activated B cells; NQO1: NAD(P)H quinone dehydrogenase 1; PI3K: phosphatidylinositol-3-kinase; Sirt1: sirtuin 1; SLC7A11: solute carrier family 7 member 11; SOD: superoxide dismutase; STAT: signal transducer and activator of transcription; TLR: Toll-like receptor; TNF: tumour necrosis factor; TrkB: tyrosine receptor kinase B.

## 7. Multi-Antioxidant Therapy for the Improvement of Clinical Outcomes

In preclinical models of IRI, the use of antioxidants has shown promising outcomes, providing insights into the underlying mechanisms. However, this advantage has not yet been effectively translated into clinical settings [3]. Using monotherapies to address the complex clinical damage caused by multifactorial mechanisms may contribute to reported discrepancies [3]. Therefore, exploring the potential effect of multi-antioxidant therapy to enhance clinical outcomes emerges as a significant possibility. 

Clinical trials have recently assessed the efficacy of Saffron aqueous extract [98] compounded by numerous antioxidants. Saffron (*Crocus sativus* L.), extensively used in herbal medicine, contains various constituents such as carotenoids, safranal, picrocrocin, crocetin, crocin, and quercetin. Saffron protects cells from OS by scavenging free radicals and inhibiting lipid peroxidation of membranes [98]. In a clinical trial evaluating Saffron’s role in reducing OS in patients with IS, promising outcomes were observed when measuring the NIHSS on days one and four [98].

Additionally, the combination of edaravone (EDV), a free radical scavenger, and borneol, a terpene and bicyclic organic compound, has been assessed [99]. The TASTE trial (Treatment of Acute Ischemic Stroke with Edaravone Dexborneol), a phase III randomised, double-blind, parallel, comparative study involving 1200 participants, demonstrated that 90-day good functional outcomes favoured treatment with edaravone dexborneol over EDV alone, particularly among female patients [100]. Table 3 summarises recent clinical trials assessing antioxidant therapy for improving outcomes after acute IS.

Oleoylethanolamide (OEA) is another compound with multiple antioxidant effects. This fatty acid has shown to reduce lipid peroxidation, prostaglandins, NO formation, and enhancing the GSH levels [101].

## 8. Conclusions

A deeper understanding of the molecular aspects of IS damage is crucial for developing novel therapeutic strategies [64]. Our understanding of the pathophysiology of IS strongly implicates OS in the mechanisms of injury, suggesting a potential protective effect through reinforcement of the antioxidant defence system. Despite numerous preclinical studies targeting OS to prevent cerebral damage, it has been challenging to successfully translate positive experimental results into clinical models.

Limited efforts have been made to explore combined therapies using antioxidants. Therefore, for future perspectives, this study encourages the assessment of strategies based on multi-antioxidant therapies. This could involve designing combinations of drugs that act at different pathways and levels. By combining several actions, an improvement in the therapeutic effects may be expected, resulting from the synergistic effects of various bioactive antioxidant molecules. This hypothesis could be tested by designing randomised, double-blinded, placebo-controlled clinical trials to optimise the known positive effects of antioxidant monotherapies. Such trials would provide valuable insights into the potential benefits of multi-oxidant approaches for treating IS.

## Figures and Tables

**Table 1:** Pathogenic molecular mechanisms during brain IRI following an IS.

Pathogenic Processes of Ischemic Stroke	Molecular Mechanisms	Ref.
Oxidative and Nitrosative Stress	Reactive oxygen species (ROS) and reactive nitrogen species (RNS) induce mitochondrial dysfunction, lipid peroxidation, and disruption of the blood–brain barrier, suppress intrinsic antioxidant effects, and induce DNA damage and cell death	[13,14]
	Nicotinamide adenine dinucleotide phosphate (NADPH) oxidase catalyses the transfer of electrons from cytosolic NADPH O_2_ at the extracellular side of the cell membrane culminating with superoxide formation	[15,16]
	Xanthine oxidase catalyses the conversion of hypoxanthine and xanthine to uric acid, producing ROS as by-products	[17,18]
	Electron leakage occurs at various points along the respiratory chain on the mitochondria, particularly at complex I and complex III, and is responsible for ROS generation	[19]
	Differential expression of neuronal nitric oxide synthase (nNOS) induces nitric oxide synthase (iNOS) and endothelial nitric oxide synthase (eNOS) with the subsequent nitric oxide production	[12]
	Nitric oxide reacts with superoxide to produce peroxynitrite	[5]
	Peroxynitrite induces direct nitrosative damage/tyrosine nitration of Keap1 (preventing Nrf2 from being activated with its antioxidant activity) and TP53-induced glycolysis and apoptosis regulator (TIGAR) with a subsequent impaired generation of NADPH	[12]
	While hypoxia-inducible factor-1 (HIF-1) activation during ischemia is generally regarded as protective by enhancing VEFG generation and stimulating angiogenesis, its role becomes more nuanced during reperfusion by promoting blood–brain barrier disruption mainly through pericytes	[20]
	Nuclear factor erythroid 2-related factor 2 (Nrf2) transcription factor upregulates multiple antioxidant response elements (AREs), conferring cytoprotective factors to the cell	[21]
Lipid Peroxidation	Fe^2+^ produces ROS and promotes lipid peroxidation	[22]
	Malondialdehyde (reactive aldehydes) can form adducts with cellular proteins and nucleic acids, contributing to cellular dysfunction	[23]
	4-Hydroxynonenal is primarily produced during the peroxidation of omega 6 polyunsaturated fatty acids	[23]
	Low-density lipoprotein (LDL) molecules could become highly oxidised to trigger macrophages activation and apoptosis	[23]
	ROS could participate in forming F2-isoprostanes through arachidonic acid oxidation	[24]
	Acyl-CoA synthetase long-chain family member 4 (ACSL4) facilitates the incorporation of polyunsaturated fatty acids into phospholipids, contributing to lipid peroxidation	[25]
	Nuclear receptor coactivator 4 (NCOA4) facilitates the selective autophagic degradation of ferritin, releasing iron and promoting the Fenton reaction, contributing to lipid peroxidation	[26]
Excitotoxicity	Glutamate activates the NMDAR, inducing a large increase in intracellular Ca^2+^ concentration, directly stimulating ROS/RNS production	[5,27]
Inflammation	Microglia display a pro-inflammatory (M1) subtype, producing multiple pro-inflammatory cytokines such as the TNFα, interferon-gamma (IFN-γ) IL-1β, IL-6, and IL-12	[28,29]
	Microglia display an anti-inflammatory (M2) subtype, expressing anti-inflammatory cytokines, including IL-10, transforming growth factor beta (TGF-β), insulin-like growth factor 1 (IGF-1), and arginase 1 (Arg1), thus participating in tissue repair and inflammation resolution	
	Janus kinase 2 (JAK2)/signal transducer and activator of transcription (STAT3) is the main signalling pathway that is responsible for activating microglia into a pro-inflammatory subtype	[30]
	NLR family pyrin domain containing 3 (NLRP3) promotes an inflammatory response and triggers neuronal pyroptosis after ischemic stroke	[31]
Blood–Brain Barrier (BBB) Disruption	Matrix metalloproteinase (MMPs) plays a role in cleaving tight junctions and degrading the extracellular matrix	[32]
	Oligodendrocytes produce large amounts of MMP-9 as a response to inflammation and oxidative stress	[33]
	Pericytes migrate away from the vasculature, thereby contributing to increased BBB permeability	[34]
	Astrocytes release vascular endothelial growth factor (VEGF), glial cell-derived neurotrophic factor, MMP, glutamate, and NO	[35,36]
	Endothelial cells induce alteration of Ca^2+^ metabolism, phospholipase-A2 activation, and production of monocyte chemoattractant protein-1	[37]
Cell Death	Autophagy: Activation by AMP-activated protein kinase (AMPK), activation by phosphatidylinositol-3-kinase (PI3K)/protein kinase B (Akt), inhibition by mammalian target of rapamycin (mTOR), activation by hypoxia-inducible factor (HIF)-1α/BCL2 interacting protein 3 (BNIP3), inhibition by the sequestration of Beclin1 by Bcl2, activation by p53, and inhibition by TIGAR	[38]
	Apoptosis: Both the intrinsic (mitochondrial) and extrinsic (death receptors) pathways are involved	[39]
	Ferroptosis: Iron-dependent accumulation of lipid peroxides and glutathione peroxidase 4 (GPX4) inactivation	[22]
	Pyroptosis: The NLRP3 inflammasome is activated by ROS generated during IRI, thus triggering pyroptosis in brain cells.	[40]
	Necroptosis: Mediated by receptor-interacting serine/threonine protein kinase-1 and -3 and mixed lineage kinase domain-like protein	[41]

ACSL4: Acyl-CoA synthetase long-chain family member 4; AMPK: AMP-activated protein kinase; Akt: protein kinase B; BBB: blood–brain barrier; BNIP3: BCL2 interacting protein 3; eNOS: endothelial NOS; GPX: glutathione peroxidase; JAK2: Janus kinase 2; HIF: hypoxia-inducible factor; iNOS: induced NOS; LDL: low-density lipoprotein; MMP: matrix metalloproteinase; mTOR: mammalian target of rapamycin; NADPH: nicotinamide adenine dinucleotide phosphate; NCOA4: nuclear receptor coactivator 4; NLRP3: NLR family pyrin domain containing 3; NMDAR: N-methyl-D-aspartate receptor; nNOS: nitric oxide synthase; NO: nitric oxide; Nrf2: nuclear factor erythroid 2-related factor 2; PI3K: phosphatidylinositol-3-kinase; TIGAR: TP53-induced glycolysis and apoptosis regulator; RNS: reactive nitrogen species; ROS: reactive oxygen species; STAT: signal transducer and activator of transcription; VEGF: vascular endothelial growth factor.

**Table 3:** Recent clinical trials assessing antioxidants for improving outcomes after an acute ischemic stroke.

Drug	Dose Frequency Length	Controlled Randomisation Blind	N (Total) N (Intervention) N (Control)	Efficacy Assessment	Main Results	Adverse Effects
Melatonin [75]	20 mg	Placebo	65	NIHSS and mRS	Higher reduction at 30 and 90 days in median NIHSS and mRS scores compared to placebo	No serious adverse events were present
	1 per day	Yes	32			
	5 days	Double	33			
Acetyl-L-Carnitine [79]	1000 mg	Placebo	69	NIHSS and mRS	Higher reduction at 90 days in NIHSS and mRS scores compared to placebo	No differences among groups
	3 per day	Yes	34			
	3 days	Double	35			
Edaravone dexborneol [100]	37.5 mg	Edaravone (alone)	1194	NIHSS and mRS	Higher reduction at 90 days in mRS score compared to placebo	No differences among groups
	2 per day	Yes	599			
	14 days	Double	595			
Saffron [98]	200 mg	Standard treatment	40	NIHSS	Higher reduction at 4 days in NIHSS scores compared to placebo	No data available
	2 per day	Yes	20			
	4 days	N/I	20			
Coenzyme Q10 [78]	100 mg	Placebo	44	NIHSS	Higher reduction at 30 days in NIHSS scores compared to placebo	No data available
	3 per day	Yes	21	mRS		
	4 weeks	Double	23	MMSE		
Oleoylethanolamide [101]	300 or 600 mg	Placebo	60	NIHSS mRS	OEA improves the inflammatory parameters, OS balance, and lipids levels	No differences among groups
	1 per day	Yes	40			
	3 days	Double	20			

NIHSS: National Institutes of Health Stroke Scale. N/I: Not informed. MMSE: Mini mental state examination; mRS: modified Rankin scale.

## Abbreviations

ACSL4	Acyl-CoA synthetase long-chain family member 4
Akt	Protein kinase B
AMPK	AMP-activated protein kinase
ARE	Antioxidant response elements
Arg1	Arginase 1
ATP	Adenosine triphosphate
ATRA	All-trans retinoic acid
ATX	Astaxanthin
BBB	Blood–brain barrier
BDNF	Brain-derived neurotrophic factor
BNIP3	BCL2 interacting protein 3
CoQ10	Coenzyme Q10
COX	Cyclooxygenases
CREB	cAMP response element-binding protein
DAMPs	Damage-associated molecular patterns
E6AP	E6-associated protein
ECs	Endothelial cells
EDV	Edaravone
eNOS	Endothelial NOS
ERK	Extracellular signal-regulated kinase
ETC	Electron transport chain
FHC1	Ferritin heavy chain 1
GPX	Glutathione peroxidase
HIF	Hypoxia-inducible factor
HMGB1	High-mobility group box protein 1
HNE	4-Hydroxy-2-nonenal
HO-1	Heme oxygenase 1
IFN	Interferon
IKKα	IκB kinase α
IKKβ	IκB kinase β
IL	Interleukin
iNOS	Induced NOS
IRF	Interferon regulatory factors
IRI	Ischemia-reperfusion injury
JAK	Janus kinase
JNK	c-Jun N-terminal kinase
Keap1	Kelch-like ECH-associated protein 1
LDL	Low-density lipoprotein
LOX	Lipoxygenases
Maf	Musculoaponeurotic fibrosarcoma
MAPK	Mitogen-activated protein kinase
MDA	Malondialdehyde
MMP	Matrix metalloproteinase
MMSE	Mini mental state examination
mRS	Modified Rankin scale
mTOR	Mammalian target of rapamycin
NADPH	Nicotinamide adenine dinucleotide phosphate
NCOA4	Nuclear receptor coactivator 4
NF-κB	Nuclear factor kappa-light-chain-enhancer of activated B cells
NIHSS	National Institutes of Health Stroke Scale
NLRP3	NLR family pyrin domain containing 3
NMDAR	N-methyl-D-aspartate receptor
nNOS	Nitric oxide synthase
NO	Nitric oxide
NOX	NADPH oxidase
NQO1	NAD(P)H quinone dehydrogenase 1
Nrf2	Nuclear factor erythroid 2-related factor 2
OEA	Oleoylethanolamide
OS	Oxidative stress
PI3K	Phosphatidylinositol-3-kinase
PRRs	Pattern recognition receptors
Prx	Peroxiredoxin
PUFA	Polyunsaturated fatty acids
RNS	Reactive nitrogen species
ROS	Reactive oxygen species
Sirt1	Sirtuin 1
SLC7A11	Solute carrier family 7 member 11
SOD	Superoxide dismutase
STAT	Signal transducer and activator of transcription
TAC	Total antioxidant capacity
TIGAR	TP53-induced glycolysis and apoptosis regulator
TLR	Toll-like receptor
TNF	Tumour necrosis factor
TP53	Tumour protein p53
TrkB	Tyrosine receptor kinase B
VEGF	Vascular endothelial growth factor
XO	Xanthine oxidase
βCAR	β-carotene
